# Basal Tumor Cell Isolation and Patient-Derived Xenograft Engraftment Identify High-Risk Clinical Bladder Cancers

**DOI:** 10.1038/srep35854

**Published:** 2016-10-24

**Authors:** K. B. Skowron, S. P. Pitroda, J. P. Namm, O. Balogun, M. A. Beckett, M. L. Zenner, O. Fayanju, X. Huang, C. Fernandez, W. Zheng, G. Qiao, R. Chin, S. J. Kron, N. N. Khodarev, M. C. Posner, G. D. Steinberg, R. R. Weichselbaum

**Affiliations:** 1 University of Chicago Medicine, Dept. of Surgery, Chicago, IL, USA; 2 University of Chicago Medicine, Dept. of Radiation and Cellular Oncology, Chicago, IL, USA; 3 The Ludwig Center for Metastasis Research, University of Chicago, Chicago, IL, USA; 4 Loma Linda University Health, Dept. of Surgery, Loma Linda, CA USA; 5 University of Illinois at Chicago, Chicago, IL USA; 6 University of California Los Angeles, Dept. of Radiation Oncology, Los Angeles, CA, USA; 7 University of Chicago, Dept. of Molecular Genetics and Cell Biology, Chicago, IL, USA

## Abstract

Strategies to identify tumors at highest risk for treatment failure are currently under investigation for patients with bladder cancer. We demonstrate that flow cytometric detection of poorly differentiated basal tumor cells (BTCs), as defined by the co-expression of CD90, CD44 and CD49f, directly from patients with early stage tumors (T1-T2 and N0) and patient-derived xenograft (PDX) engraftment in locally advanced tumors (T3-T4 or N+) predict poor prognosis in patients with bladder cancer. Comparative transcriptomic analysis of bladder tumor cells isolated from PDXs indicates unique patterns of gene expression during bladder tumor cell differentiation. We found cell division cycle 25C (CDC25C) overexpression in poorly differentiated BTCs and determined that CDC25C expression predicts adverse survival independent of standard clinical and pathologic features in bladder cancer patients. Taken together, our findings support the utility of BTCs and bladder cancer PDX models in the discovery of novel molecular targets and predictive biomarkers for personalizing oncology care for patients.

An estimated 77,000 patients will be diagnosed with bladder cancer in the United States in 2016, with over 16,000 dying of their disease, making bladder cancer the 4^th^ most common cause of cancer-related death among men^1^. Cystectomy remains at the core of treatment for invasive bladder cancer. Recent findings also suggest a clinical benefit from systemic therapy^2^,^3^,^4^,^5^. However, a substantial proportion of patients fail standard therapy. While there have been advances toward risk stratification and personalization of oncologic therapies in many cancers, there is a critical need to identify new molecular markers for the classification and treatment of high-risk bladder cancer patients.

Volkmer *et al*. identified three bladder tumor cell differentiation states (basal, intermediate and differentiated) based on the expression of keratins 14, 5 and 20, which are associated with the expression of cell surface markers CD90, CD44 and CD49f: basal or triple positive (CD90+/CD44+/CD49f+), intermediate or double positive (CD90−/CD44+/CD49f+), differentiated or single positive (CD90−/CD44−/CD49f+) and fully differentiated or triple negative cells (CD90−/CD44−/CD49f−). Notably, the least differentiated cells within a tumor population demonstrated tumor-initiating capacity^6^. While this study correlated the expression of keratin 14 with patient outcomes, it is unknown whether cell surface marker patterns could serve as a direct measure of basal tumor cells (BTCs) in patient bladder cancer samples.

We hypothesized that patients whose tumors harbored poorly differentiated or triple positive BTCs would have a heightened malignant potential and thus be at higher risk for treatment failure. We report a strategy for the immediate isolation of BTCs from patient tumors at the time of cystectomy, which demonstrated an association of BTCs with an increased risk for death in early stage tumors. We also report the expansion of BTCs using patient-derived xenografts (PDXs) and determined that PDX engraftment correlates with poor survival in locally advanced bladder cancers. By performing comparative gene expression analysis, we characterized bladder tumor cell subpopulations consisting of single, double, and triple positive cells reflecting progression of bladder cancer dedifferentiation^6^. We report cell division cycle 25C (CDC25C) is overexpressed in poorly differentiated bladder tumor cells (BTCs) and predictive of adverse survival after radical cystectomy independent of clinical and pathologic factors in three bladder cancer data sets comprising approximately 400 patients. Taken together, these findings support the immediate detection of basal bladder cancer cells in combination with PDX models to improve patient risk stratification and identify novel therapeutic targets.

## Results

### Isolation of Patient-Derived Basal Bladder Tumor Cells

**Figure 1:**
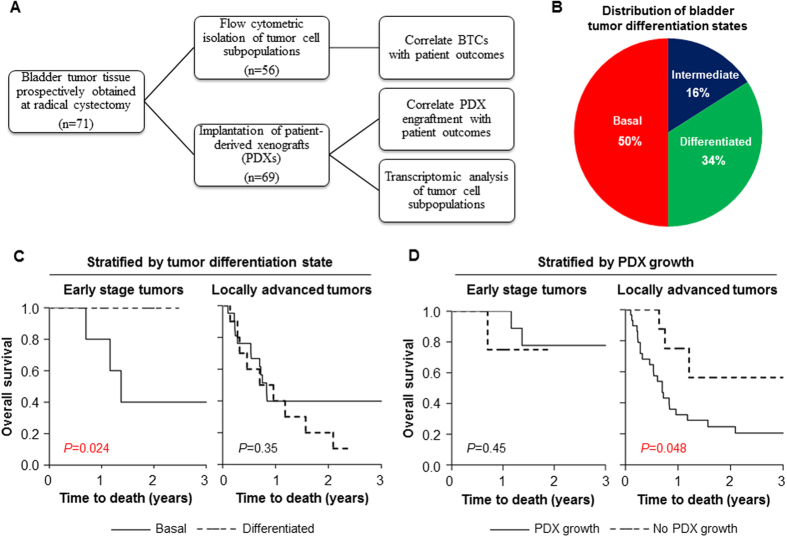
Prognostic value of basal tumor cell isolation and patient-derived xenograft engraftment in clinical bladder cancers. (**A**) Schematic for prospective collection of patient-derived bladder cancers at the University of Chicago Medical Center. (**B**) Distribution of bladder tumor differentiation states (n = 56) based on flow cytometric isolation of basal or triple positive (CD90+/CD44+/CD49f+), intermediate or double positive (CD90−/CD44+/CD49f+) and differentiated or single positive (CD90−/CD44−/CD49f+) bladder tumor cells. (**C**) Kaplan-Meier curves comparing overall survival for patients with basal and differentiated tumors in early stage ([pathologic] pT1-T2 and N0 [lymph node-negative]; n = 15) and locally advanced (pT3-T4 or N + [lymph node-positive]; n = 33) tumors. (**D**) Kaplan-Meier curves comparing overall survival for patients stratified by PDX engraftment in early stage and locally advanced tumors. BTC, basal tumor cell. PDX, patient-derived xenograft. Time to death was calculated as the interval between radical cystectomy and date of death or last follow-up. *P*-values were determined using log-rank tests.

Bladder tumor specimens (n = 71) were prospectively collected from patients undergoing radical cystectomy for bladder cancer at the University of Chicago Medical Center (Table 1). Fresh bladder tumor tissue fragments from cystectomy specimens were immediately analyzed by flow cytometry and categorized into differentiation states based on the expression of cell surface markers as previously described^6^ (Fig. 1A). We classified tumors based on the least differentiated detectable cell type, as was determined by expression of CD90, CD44, and CD49f, with a lower limit of flow cytometric detection of 0.1% of the total cell population. Of 56 evaluable tumors, 28 (50%) were triple positive (basal), 9 (16%) were double positive (intermediate) and 19 (34%) were single positive (differentiated) (Fig. 1B). No tumor was classified as triple negative (fully differentiated). Basal tumor cells, when present, comprised ~1.3% (median) of all tumor cells (interquartile range: 0.2–3%), whereas fully differentiated cells comprised ~77% (median) of tumor cells (interquartile range: 50–90%).

We hypothesized that patient tumors harboring a detectable population of basal bladder tumor cells would have an increased malignant potential, and, therefore, would be associated with a higher risk for death after treatment. In this regard, we found that locally advanced tumors (pathologic or pT3-T4 and/or positive for lymph node metastasis (N+)) were more likely to harbor basal cells as compared to early stage tumors (79% vs. 53%, *P* = 0.06). In patients with early stage disease (pT1-T2 and N0), the presence of basal tumor cells on immediate flow cytometry was a marker for decreased overall survival after cystectomy (3-year overall survival for basal vs. differentiated: 40% vs. 100%, log-rank *P* = 0.024; Fig. 1C). These findings suggested that poorly differentiated BTCs are highly prevalent in locally advanced tumors and could serve as a marker for poor prognosis in early stage bladder cancers.

### Engraftment of Patient-Derived Xenografts Predicts Poor Survival in Locally Advanced Bladder Cancer

In parallel studies, fresh bladder tumor tissue fragments were subcutaneously injected into the flanks of non-obese diabetic (NOD), homozygous severe combined immunodeficiency (SCID) mice based on prior studies demonstrating excellent PDX engraftment rates in NOD/SCID mice^7^,^8^. From August 2012 to May 2015, 69 patient-derived primary bladder tumors were implanted for PDX establishment, of which 42 (61%) successfully engrafted within approximately 1–3 months after injection (Fig. 1A). Both muscle-invasive and superficial bladder cancers demonstrated engraftment (38 of 60 invasive tumors (63%); 4 of 9 superficial tumors (44%)). Histologically PDX tumors appeared similar to corresponding original patient tumors (Figure S1). There were no clinical or pathologic features predictive of successful PDX engraftment (Table S1). In addition, PDX engraftment was unaffected by bladder cancer differentiation state (Table S1).

Among patients with locally advanced tumors and/or lymph node metastasis, PDX engraftment was associated with an increased risk for death after treatment (Hazard ratio [HR] = 3.0; 95% CI: 1.05–12.95, *P* = 0.040) when compared with non-PDX-engrafting patients (Fig. 1D). In contrast, overall survival was independent of PDX engraftment in patients with early stage disease (Fig. 1D). Importantly, PDX engraftment and BTC detection could be integrated to classify patients with distinct outcomes. In a multivariate Cox proportional hazard analysis for overall survival accounting for tumor stage and lymph node involvement, PDX engraftment and the presence of basal tumor cells were independently associated with 6.2-fold (95% CI: 1.6–41.1, *P* = 0.0059) and 2.7-fold (95% CI: 0.97–8.30, *P* = 0.057) increased risks of death. These findings suggest that BTCs and PDX engraftment provide complementary prognostic information for clinical bladder cancers.

### Comparative Gene Expression Analysis of Basal Bladder Tumor Cells

**Figure 2:**
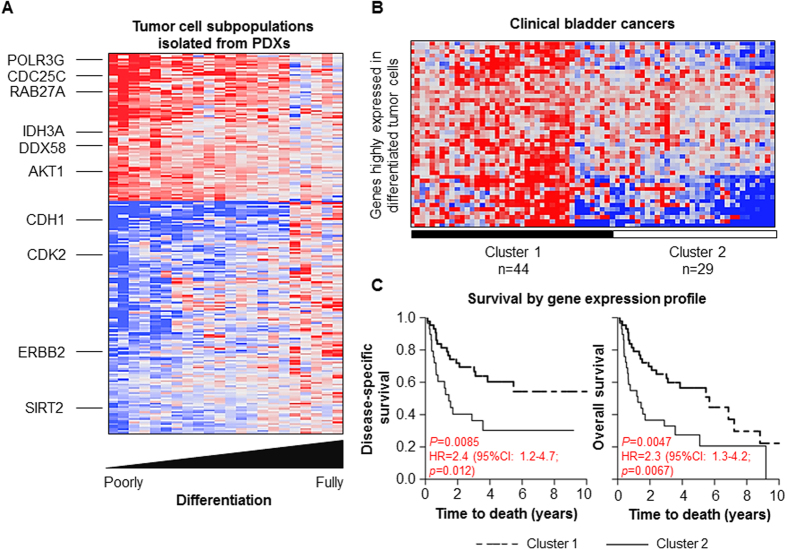
Gene signature associated with bladder tumor cell differentiation and poor prognosis in patients with bladder cancer. (**A**) Heat map showing expression of 88 correlated and 136 anti-correlated genes associated with tumor cell differentiation. Five independent PDXs were flow sorted into respective tumor cell subpopulations and were available for microarray gene expression analyses. Red color indicates high expression, while blue color indicates low expression. (**B**) Hierarchical clustering of differentially expressed tumor cell differentiation-associated genes identified two groups of patients from the MD Anderson Cancer Center data set^9^ (**C**). Kaplan-Meier survival curves for patient clusters stratified by tumor cell differentiation-associated gene expression. Time to death was calculated as the interval between radical cystectomy and date of death or last follow-up. *P*-values were determined using log-rank tests. HR, hazard ratio; 95% CI, confidence interval; *P*-value determined using univariate Cox proportional hazard analysis.

We utilized PDX models to facilitate the expansion of BTCs for subsequent molecular analysis. PDX engraftment allowed amplification of rare basal cell populations at similar frequencies when compared to the original donor tumor, while maintaining a heterogeneous tumor cell population (Table S2). Utilizing comparative transcriptomic analysis, we investigated genes associated with the four stages of bladder tumor cell differentiation. We hypothesized that BTCs progress through a step-wise differentiation process, and, therefore, we examined genes which correlated or anti-correlated with the progression of BTC differentiation from triple positive to triple negative states. This analysis resulted in 88 correlated genes and 136 anti-correlated genes in BTCs (Fig. 2A and Table S4). Among the genes with elevated expression in BTCs included effectors involved in oncogenesis (e.g. RAB27A, member of the RAS oncogene family; AKT1, RAC-alpha serine/threonine-protein kinase), cell cycle progression (e.g. CDC25C, Cell Division Cycle 25C), metabolism (e.g. IDH3A, isocitrate dehydrogenase; PDHA1, pyruvate dehydrogenase; PFKP, phosphofructokinase) and nucleic acid sensing (e.g. DDX58, DEAD box polypeptide 58; DDX54, DEAD box polypeptide 54; POLR3C/3G/3K, RNA polymerase III polypeptide C/G/K). In contrast, among the genes suppressed in BTCs were epithelial markers previously described in the “luminal” subtypes of bladder cancer (e.g. CDH1, E-cadherin; ERBB2, receptor tyrosine kinase 2, a known oncogene)^9^. These results demonstrated that poorly differentiated BTCs, as compared to fully differentiated tumor cells, exhibit distinct patterns of gene expression.

### BTC Gene Expression is Prognostic of Clinical Outcome

We evaluated whether BTC gene expression patterns correlated with poorer prognosis in a publicly available clinical data set of human bladder cancer^9^. We found BTC gene expression patterns distinguished patient tumors into two groups with significant differences in disease-specific and overall survivals (Fig. 2B,C). Based on these findings, we selected CDC25C, a top-ranked gene correlated with tumor cell dedifferentiation, for further validation. CDC25C permits cell entry into mitosis by dephosphorylation of CDK1^10^. Notably, CDC25 overexpression has been reported in a large number of human cancers^11^; however, the significance of CDC25C overexpression in human cancers and its association with adverse clinical outcomes remain poorly understood.

**Figure 3:**
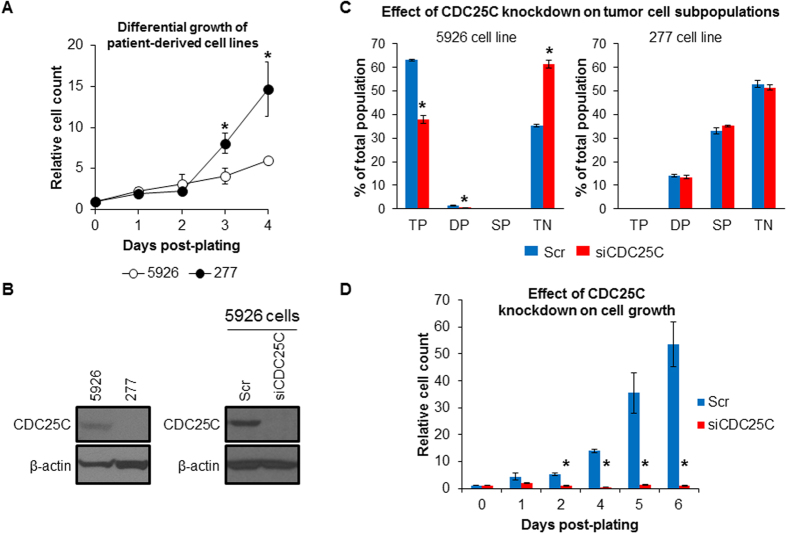
CDC25C promotes tumor cell dedifferentiation and growth. (**A**) Differential growth of 5926 (predominantly basal cells) and 277 (predominantly fully differentiated cells) cell lines *in vitro*. Cell count was manually determined using a hemocytometer for four biological replicates per sample. Relative cell count was determined by normalization to day 0 values. (**B**) Western blot analysis of CDC25C protein expression in parental 5926 and 277 cell lines and in 5926 cells after treatment with siRNA against CDC25C (siCDC25C) or a scrambled control (Scr). β-actin protein level served as a loading control. (**C**) Flow cytometric analysis of bladder tumor cell subpopulations after treatment of 5926 and 277 cells with siRNA against CDC25C or a scrambled control. TP, triple-positive (basal); DP, double-positive (intermediate); SP, single-positive (differentiated); TN, triple-negative (fully differentiated). (**D**) Effect of CDC25C gene suppression on 5926 cell growth. Relative cell count was determined by normalization to day 0 values. Error bars denote standard error of mean. *P*-values were determined using 2-tailed Student’s *t*-test. Asterisks denote *P*-values ≤ 0.05.

We generated cell lines from patient-derived tumors using serial dilution of cell suspensions and evaluated BTC content of the derived cell lines using flow cytometry (Table S3). This procedure led to the development of two cell lines of particular interest: 5926 clone 2 (herein referred to as 5926) enriched for basal cells (triple-positive: 66%; double-positive: 7.8%; single-positive: 6.3%; triple-negative: 19.7%) and 277 clone 7 (herein referred to as 277) enriched for differentiated triple negative cells (triple-positive: 0%; double-positive^2^^2^: 0%; single-positive: 1.1%; triple-negative: 98.9%). 5926 cells established *in vitro* proliferated at slower rates than 277 differentiated cells (Fig. 3A). Western blot analysis of 5926 and 277 cell lines confirmed overexpression of CDC25C in 5926 cells (Fig. 3B). In addition, CDC25C gene suppression in 5926 cells using siRNA significantly decreased the percentage of triple positive cells from 66% to 38% (*P* *=* 0.0043, Fig. 3C) and arrested tumor cell growth (Fig. 3D). In contrast, suppression of CDC25C in 277 cells had no measurable effect on the frequencies of tumor cell subpopulations (Fig. 3C). These findings suggested that overexpression of CDC25C contributes to the malignant phenotype of poorly differentiated basal bladder tumor cells.

### CDC25C Overexpression Identifies Poor Prognosis Human Bladder Cancer Patients

**Figure 4:**
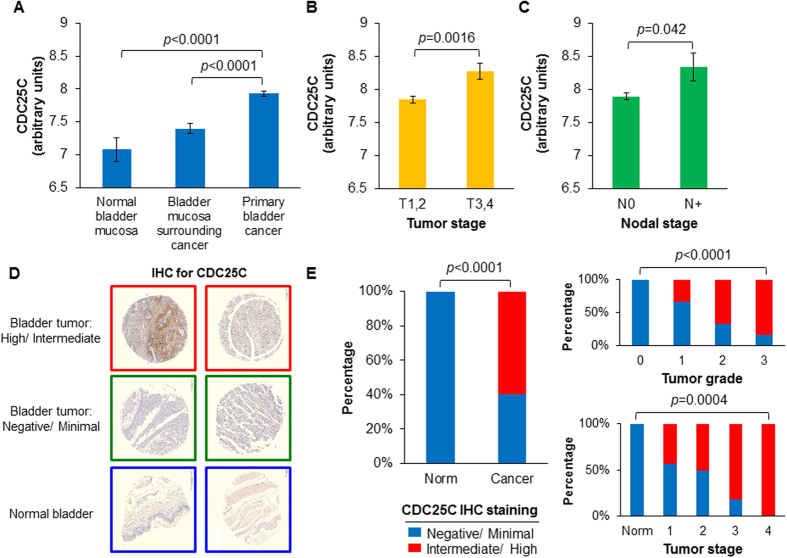
CDC25C overexpression is prognostic in clinical bladder cancers. (**A**) CDC25C gene expression in clinical specimens derived from normal bladder mucosa (n = 10), bladder mucosa surrounding cancer (n = 58) and primary bladder cancers (n = 165). Gene expression values were estimated by normalized microarray signal intensity in the Chungbuk Cancer Center data set^12^. CDC25C gene expression differences in early (T1-2, n = 135) vs. advanced (T3-4, n = 30) primary bladder cancers (**B**) and lymph node-negative (N0, n = 149) vs. lymph node-positive (N+, n = 15) bladder tumors (**C**). *P*-values were determined using 2-tailed Student’s *t*-test. (**D**) Representative immunohistochemical (IHC) staining for CDC25C in a bladder cancer tissue microarray demonstrating differences in staining intensity across bladder tumors and normal bladder. IHC staining was categorized as follows: “Negative” = rare positive cells, <5%; “Minimal” = <25% highly positive cells or majority of cells with <25% stained nucleus; “Intermediate”=25–75% highly positive cells or majority of cells with 25–75% stained nucleus; “High” = >75% highly positive cells. (**E**) Differences in intermediate/high CDC25C IHC staining in comparisons of normal bladder mucosa (Norm, n = 10) and primary bladder tumors (Cancer, n = 40) [Left] and by tumor grade [Right, top] and overall tumor stage [Right, bottom]. *P*-value determined using χ^2^ tests.

In the context of these findings, we examined CDC25C gene expression in clinical samples of human bladder cancer^12^. We found a step-wise increase in CDC25C expression from normal bladder mucosa to bladder mucosa surrounding cancer and finally to primary bladder cancers (Fig. 4A). In addition, CDC25C expression was elevated in locally advanced pT3-T4 tumors (Fig. 4B) and associated with lymph node metastasis (Fig. 4C). Immunohistochemical analysis of CDC25C protein expression using a human bladder cancer tissue microarray demonstrated a spectrum of nuclear CDC25C expression ranging from highly expressed in nearly all cells to limited expression in few cells (Table S5 and Fig. 4D). In comparison, normal bladder tissue exhibited little or no expression (Fig. 4D). Also, CDC25C protein expression was greater in high grade and advanced stage tumors as compared to low grade and early stage tumors (Fig. 4E). Given that CDC25C gene expression increases with the degree of dedifferentiation, these immunohistochemical findings suggest that high tumoral expression of CDC25C protein signifies an increased extent of undifferentiated bladder tumor cells rather than exclusively triple-positive basal cells.

Several groups have proposed classification systems to identify intrinsic subtypes of bladder cancer. Recently, Aine *et al*.^13^ examined the interrelations of three proposed gene expression-based classification systems. The authors identified consensus cluster subgroups that converged on two major molecular subtypes – basal and luminal – distinguished by squamous versus urothelial differentiation. In this context, we examined whether CDC25C, a marker for bladder dedifferentiation, is overexpressed in basal subtypes as compared to luminal subtypes. In the MD Anderson Cancer Center (MDACC) dataset of Choi *et al*.^9^ and the Lund University Hospital dataset of Sjodahl *et al*.^14^ we detected an overexpression of CDC25C in the basal tumor subtypes as compared to luminal tumors (Figure S2). In addition, CDC25C gene expression correlated with the degree of dedifferentiation across the respective subgroups within each classification system. Taken together, these findings demonstrated that CDC25C overexpression is characteristic of un-differentiated basal tumor cells, as well as basal tumor subtypes.

In multivariate Cox proportional hazard analyses controlling for pathologic tumor covariates, CDC25C expression was an independent risk factor for death after radical cystectomy in three independent data sets (n = 396) of human bladder cancer (Table 2)^9^,^12^,^14^. We hypothesized that CDC25C expression could also predict benefit after chemotherapy given its established role in cell cycle regulation. In a meta-analysis of two independent data sets (n = 38) of patients who received platinum-based chemotherapy after radical cystectomy, CDC25C overexpression was an independent risk factor for improved survival (Table S6). These findings demonstrate that CDC25C overexpression predicts adverse prognosis after radical cystectomy, but also potentially identifies a subset of higher risk patients who may derive benefit from platinum-based chemotherapy.

## Discussion

We demonstrate the feasibility of immediate flow cytometric detection of BTCs from bladder cancer patients undergoing radical cystectomy and its association with adverse survival in early stage bladder cancer. In conjunction, we identify PDX engraftment as predictive of poor outcomes among patients with advanced disease. Utilizing PDX models, we expanded BTCs to evaluate transcriptomic patterns associated with tumor cell dedifferentiation. Importantly, we found CDC25C overexpression to be a key feature of the BTC phenotype and show that CDC25C expression is an independent risk factor for death after surgery in clinical bladder cancers.

Our study is unique in that we combined BTCs immediately identified from surgical specimens and PDX engraftment to construct a multivariate risk model for death in bladder cancer. Previous studies have reported an association between PDX engraftment and outcome in breast, lung, pancreatic and ovarian cancers^7^,^15^,^16^,^17^. However, our study is the first to demonstrate a differential prognostic effect of detectable BTCs and PDX engraftment based on pathologic tumor features. Additionally, tumors harboring BTCs exhibited more aggressive pathologic features, which is consistent with prior studies demonstrating an association between BTCs and adverse prognoses in bladder and other cancer types^6^,^18^,^19^. Future studies are required to evaluate whether the absence of basal cells on immediate flow cytometric analysis of diagnostic transurethral bladder tumor specimens may spare some patients the need to undergo radical cystectomy.

To our knowledge, this study describes the largest cohort of bladder cancer PDXs to-date. Our PDX engraftment rate of 61% is among the highest reported^7^,^15^,^16^. An appeal of PDX studies is the ability of the resulting tumor xenograft to recapitulate the behavior of a patient’s tumor, allowing for more accurate assessments of tumor behavior *in vivo* and molecular properties^20^,^21^,^22^. In addition, numerous studies have applied PDX models for drug development, phase 2-style drug testing trials and biomarker discovery^23^,^24^. A unique aspect of our study is the utilization of PDX models to isolate basal tumor cells, which are typically difficult to isolate in culture since the undifferentiated cells exist in limited proportions and are known to differentiate under *in vitro* conditions.

Through comparative transcriptomic profiling, we evaluated gene expression patterns correlating with the degree of bladder tumor cell differentiation. This provided a spectrum of transcriptomic changes reflecting the various stages of tumor cell differentiation. Our analysis identified several gene categories associated with BTCs, including oncogenes, cell cycle regulators, metabolic pathways and nucleic acid sensors. Among these, a single gene was closely associated with basal cells and was further validated in this report. However, further study into the genes identified by this analysis may identify additional molecules responsible for tumor development, with potential targets for directed cancer therapy. A recent genomic analysis of non-muscle-invasive bladder cancer classified tumors based on patterns of gene expression similar to those seen in muscle-invasive bladder cancer^25^. Notably, the more aggressive tumor classes expressed CDC25A and other markers reported by Volkmer *et al*., which formed the basis of our BTC identification scheme.

We also report the clinical significance of CDC25C overexpression in bladder cancer as a determinant of poor prognosis. We propose CDC25C is differentially overexpressed in basal tumor cells and associates with tumor cell dedifferentiation. Importantly, CDC25C is also overexpressed in basal tumor subtypes identified by existing classification systems proposed for clinical bladder cancers. By extending our finding to clinical data sets of approximately 400 patients with bladder cancer, we confirmed an association of CDC25C expression with poor survival in bladder cancer patients. The CDC25 phosphatases have been hypothesized as important regulators of cancer biology due to their critical role in cell cycle regulation^26^. Many cancers have been reported to overexpress CDC25A and CDC25B, but CDC25C has not previously been associated with cancer outcome^27^. We also noted that patients with tumors harboring elevated CDC25C expression who did not receive chemotherapy were at a higher risk for death when compared to patients with low CDC25C expression. Interestingly, administration of chemotherapy abrogated the adverse prognosis associated with CDC25C overexpression. A salient translational finding is that patients with detectable triple positive BTCs and/or overexpression of CDC25C might benefit from the addition of platinum-based chemotherapy treatment, independent of their tumor or nodal staging.

In summary, we demonstrate the utility of immediate flow cytometry to identify basal tumor cells derived from surgical specimens and patient-derived xenograft growth as potential predictive features of bladder cancer. Importantly, we were able to immediately isolate triple positive BTCs from patient tumors to enhance our capability to identify patients at increased risk. Additionally, we reported the utility of both BTCs as well as patient-derived xenografts in the discovery of biomarkers in human bladder cancer. We characterized CDC25C as a novel prognostic marker in human bladder cancer, which may prove useful for the selection of patients at high risk for treatment failure. These findings constitute a critical step toward the elucidation of the biology of tumor differentiation in bladder cancer and improvement of therapy selection for bladder cancer patients.

## Materials and Methods

### Study Design

The objectives of this study were the following: (1) to identify the role of basal tumor cells in bladder cancer, and determine whether basal tumor cell detection was associated with increased risk of death after cystectomy, (2) to evaluate the role of patient-derived xenografts as a prognostic tool in bladder cancer, and (3) to characterize molecular markers associated with poor survival in bladder cancer. We hypothesized the following: (1) basal tumor cells are predictive of poor survival when immediately identified in bladder cancers, (2) patient-derived xenografts associate with poor survival in bladder cancer, as has been reported in other cancer types, and (3) analyzing basal tumor cells will identify molecular markers associated with tumor development. Hypotheses which resulted from the initial experiments included: (1) CDC25C is a marker associated with basal tumor cells, and (2) CDC25C overexpression is a marker for poor survival in bladder cancer.

The study was designed to collect all patient data available through the tissue collection protocol as approved by the University of Chicago Institutional Review Board (IRB). All patients who consented to tissue collection were included in the tissue and data collection arm of the study. All methods and experiments were performed in accordance with the University of Chicago Institutional guidelines and regulations. Samples found to be of non-urothelial origin upon final pathology were excluded; this included one patient with rhabdomyosarcoma of the bladder. All other specimens were included in the study. The experimental design was a controlled laboratory experiment, with specific components described below.

### Patient Data

A total of 71 patients treated for bladder cancer with radical cystectomy at the University of Chicago were included in the study. All patients provided informed consent for inclusion in the study as approved by the University of Chicago Institutional Review Board. Clinical and pathological data were entered into a prospectively maintained database using the University of Chicago Redcap online data collection platform. All experimental protocols were approved by the University of Chicago IRB (#15550B).

### Bladder Tumor Tissue Processing

Tissue was received from pathology in dissociation medium (M199 medium with 20mM Hepes, 1% penicillin/streptomycin, 1% Pluronic-F68). A portion of the tumor was separated for xenograft injection. The remaining tissue was disaggregated in Miltenyi gentleMACS tube tissue dissociator, then enzymatically using DNase and Liberase at 37 °C for 45 minutes. The single-cell suspension was filtered through a 70-μm filter, treated with red blood cell lysis buffer, and washed with PBS.

### Flow Cytometric Analysis

Single-cell suspensions of patient tumor tissue were stained using the following antibodies: phycoerythrin (PE)-conjugated anti-CD44 (550989 BD Pharmigen), PE-Cy7-conjugated anti-CD90 (561558 BD Pharmigen), APC-conjugated anti-CD49f (313616 BioLegend), a lineage mixture containing Pacific-blue conjugated anti-CD45 (304022 BioLegend), anti-CD31 (303114 BioLegend) and live/dead stain (L34964 LIVE/DEAD Fixable Violet Dead Cell Stain Kit, Life Technologies). Patient-derived xenograft tissue was stained using the above antibodies with the addition of Pacific-blue conjugated anti-CD45 (103126 BioLegend), anti-CD31 (102422 BioLegend), and H2K^d^ (116616 BioLegend). Flow cytometry was performed using the BD LSRFortessa and BD LSR-II and cells were sorted using BD FACSAriaIII under 20psi using a 100 μm nozzle.

### Implantation of Tissue Fragments in NOD/SCID Mice

Animal experiments were approved by the University of Chicago Institutional Animal Care and Use Committee. Human bladder tumor fragments and adjacent normal bladder tissue (1 mm^3^ pieces, 200 μL of each) were injected subcutaneously using a 12-gauge precision trocar into the backs of 6- to 10-week-old NOD/SCID mice under inhalational anesthesia. The wounds were closed with 3 M Vetbond tissue adhesive. Tumors were measured twice weekly to assess for tumor growth. Mice were sacrificed when the tumor reached approximately 2 cm^3^, if they appeared to be suffering (e.g. due to tumor ulceration) or after 6 months if no tumor was detected. Subsequent xenograft tumors were processed according to the protocols described above.

### Generation of Cell Lines from Patient Tissue

Tumor tissue was received and processed as described above. A single-cell suspension was serially diluted to a concentration of less than 1 cell per well in a 96 well culture plate. The plates were then monitored for growth. Resulting clones were expanded, harvested, and frozen in stock vials for further experiments.

### RNA Extraction and Array Preparation

Flow sorted cells as well as a sample of cells from the bulk tumor (1 × 10^6^ cells) were collected into PBS with RNAsecure buffer, centrifuged and the supernatant discarded. The cell pellet was re-suspended directly using 100 μL of Trizol LS reagent. 25 μL chloroform was added, and then centrifuged at 12,000 g for 15 minutes at 4 °C. The aqueous phase was collected, 100 μL isopropanol was added and mixed, and the solution stored at 4 °C overnight. The samples were then centrifuged at 15,000 rpm for 30 minutes at 4 °C, and the supernatant decanted. The RNA pellet was then dissolved in 30 μL of RNase-free water with RNAsecure, and samples were evaluated for quality control by the University of Chicago Genomics Core. RNA samples with acceptable quality in all cell populations (triple positive, double positive, single positive, triple negative and total tumor) were assayed in duplicate using Illumina HumanHT-12 v4 Expression BeadChip microarrays at the University of Chicago Genomics Core Facility. Gene expression data have been deposited in Gene Expression Omnibus (accession number is pending).

### Bioinformatic Analysis

Raw array data was processed using Illumina Genome studio v2011.1. Processed data were analyzed using Partek Genomics suite v6.6. Pearson correlation analysis was performed to identify genes significantly correlated with tumor cell differentiation from triple positive (assigned a value of 3) to double positive (assigned a value of 2) to single positive (assigned a value of 1) and finally to triple negative (assigned a value of 0) cells. Significant genes (p < 0.05) with absolute values of correlation coefficients ≥0.5 were evaluated using Ingenuity Pathway Analysis (IPA, Redwood City, CA).

Clinical cancer data sets and corresponding microarray gene expression data were downloaded from Gene Expression Omnibus (GEO) using the following accession numbers: MD Anderson (GSE48277), Chungbuk (GSE13507) and Lund (GSE32894). Normalized microarray probe intensity values were retrieved for each respective study and used as a surrogate for gene expression measurements. Stage IV patients with metastatic disease or those patients without a specified stage or treatment were excluded from analysis. All statistical analyses were performed with JMP 9.0 (SAS Institute Inc.).

### Tissue Microarray and Immunohistochemistry

A bladder cancer and normal tissue array was obtained from a commercial vendor (BL1002a US Biomax, Inc.). Immunohistochemistry staining was performed by the University of Chicago Human Tissue Resource Center Core. Anti-CDC25C antibody was used at a concentration of 1:2000 (ab32444 Abcam Inc.). DAB was used as a secondary reporter molecule.

### Western Blot Analysis

Total cellular protein was extracted and normalized as previously described^28^. Equal amounts of protein were loaded per well (25–30 μg). Proteins were separated on 7.5–10% SDS-PAGE and transferred to polyvinylidene difluoride (PVDF) membranes. β-actin antibody was used for loading controls (sc-47778 Santa Cruz). Anti-CDC25C antibody was used for protein detection (ab32444 Abcam Inc.).

### Gene Knockdown using siRNA

Gene knockdown was performed using the following siRNAs: (Dharmacon) CDC25C (D-003228-05, D-003228-06, D-003228-07, D-003228-08) and non-targeting control (D-001206-13-05). Transfection was performed using Opti-MEM media, GE Dharmacon DharmeFECT 1 transfection reagent (Dharmacon T-8001-02). 25 nM of each individual siRNA was added to 200,000 cells per well in a 6-well plate. The transfection condition was incubated at 37 degrees Celsius prior to downstream assays, or for 48 hours prior to Western blot confirmation of knockdown. One siRNA knockdown confirmed on Western blot analysis (Dharmacon siRNA D-003228-08) was used for downstream assays. Flow cytometry was performed using cells harvested after 48 hour of incubation with siRNA.

### Statistics

All statistical analyses were performed with JMP 9.0 (SAS Institute Inc.). Student’s *t* tests were used to calculate differences in continuous covariates and χ^2^ tests were used to calculate differences in categorical covariates between patient groups. Overall survival was calculated as the time from surgical resection until the date of death or last follow-up. The Kaplan-Meier method was applied to calculate estimates for survival probabilities. The Log-rank test was used to compare survival curves for differences in disease-specific and overall survival between patient groups. Cox proportional hazard models were utilized to examine the differential prognostic effects of predefined covariates. Statistical significance was determined using a *P* < 0.05.

## Additional Information

**How to cite this article**: Skowron, K. B. *et al*. Basal Tumor Cell Isolation and Patient-Derived Xenograft Engraftment Identify High-Risk Clinical Bladder Cancers. *Sci. Rep.* **6**, 35854; doi: 10.1038/srep35854 (2016).

## Supplementary Material

Supplementary Information

Supplementary Figure S1

Supplementary Figure S2

## Figures and Tables

**Table 1:** Baseline patient and tumor characteristics of bladder cancer patients treated with radical cystectomy.

Characteristic	No. of Patients (%)
Total number of patients	71
Mean age ± SD (years)	69.5 ± 10.7
Sex	
Male	50 (70.4%)
Female	21 (29.6%)
Race	
Caucasian	51 (71.8%)
African-American	12 (16.9%)
Asian	5 (7%)
Hispanic	1 (1.4%)
Other/Unknown	2 (2.8%)
Smoking History	
Past smoker	50 (70%)
Current smoker	8 (11%)
Never smoker	12 (17%)
Unknown	1 (1.4%)
**Pathology**	
Pathologic Tumor Stage	
Ta, T1, Tis	9 (12.7%)
T2	11 (15.5%)
T3	40 (56.3%)
T4	11 (15.5%)
Pathologic Nodal Stage	
N0	42 (59.2%)
N1	7 (9.9%)
N2	22 (31%)
Histology	
Urothelial/Papillary	57 (80.3%)
Squamous differentiation	9 (12.7%)
Other	5 (7%)
Positive surgical margin	16 (22.5%)
Tumor multifocality	22 (31%)
Metastatic disease at diagnosis	0 (0%)
**Additional Treatment**	
Chemotherapy	28 (39.4%)
Pre-operative	5 (7%)
Post-operative	18 (25.4%)
Pre- and post-operative	5 (7%)
**Outcome**	
Median follow-up in months (range)	14.9 (1–82)

“Other” histology includes small cell and sarcomatoid subtypes. Follow-up time is the time interval between diagnosis of bladder cancer to last patient encounter or date of death.

**Table 2:** Multivariate Cox proportional hazard analysis of CDC25C gene expression for bladder cancer patients undergoing radical cystectomy.

Data set	Covariate	HR	95% CI	*P*-value
MD Anderson (n = 37)	CDC25C	2.26	1.04–5.40	0.0382
	T3,4 vs. T1,2	4.08	0.98–28.7	0.0536
	N+ vs. N0	1.2	0.39–4.22	0.7594
Chungbuk (n = 135)	CDC25C	1.68	1.03–2.70	0.0396
	T3,4 vs. T1,2	6.98	2.56–16.9	0.0004
	N+ vs. N0	1.43	0.37–4.90	0.584
Lund (n = 224)	CDC25C	2.27	1.05–4.77	0.038
	T3,4 vs. T1,2	5.86	1.92–14.9	0.0037

Pathologic tumor and nodal stages and CDC25C gene expression were included as covariates for bladder cancer patients undergoing radical cystectomy without perioperative chemotherapy. Tumor stage and nodal stage were considered binary variables; CDC25C gene expression was considered a continuous variable. HR, hazard ratio; 95% CI, confidence interval. Nodal staging was excluded from analysis of the Lund data set due to insufficient data.
