# Supplementary material for: Basal Tumor Cell Isolation and Patient-Derived Xenograft Engraftment Identify High-Risk Clinical Bladder Cancers
Source: Sci Rep. 2016 Oct 24;6:35854. doi: 10.1038/srep35854 (PMC5075783; doi:10.1038/srep35854)
Supplement: Supplementary Information [file srep35854-s1.doc]

**Supplementary Materials: Basal Tumor Cell Isolation and Patient-Derived Xenograft Engraftment Identify High-Risk Clinical Bladder Cancers**

**Authors:** K. B. Skowron1,^, S. P. Pitroda2,3,^, J. P. Namm^1,4^, O. Balogun^2,3^, M. A. Beckett^2,3^, M. L. Zenner^2,3,5^, O. Fayanju^2^, X. Huang^2,3^, C. Fernandez^2,3,5^, W. Zheng^2,3^, G. Qiao^2,3^, R. Chin^2,3,6^, S. J. Kron^7^, N. N. Khodarev^2,3^, M. C. Posner^1^, G. D. Steinberg^1^, R. R. Weichselbaum^2,3,*^

**Table S1.** Baseline patient and tumor characteristics according to ability of engraftment of patient-derived xenografts

**Table S2.** Tumor cell subpopulations in patient specimens and over successive generations of patient-derived xenografts

**Table S3.** Categorization of patient-derived bladder tumor cell lines by flow cytometry

**Table S4.** Genes correlating or anti-correlating with bladder tumor cell differentiation

**Table S5.** Immunohistochemical staining for CDC25C in a bladder cancer tissue microarray

**Table S6.** Meta-analysis of multivariate hazard ratio of CDC25C expression accounting for tumor and nodal staging for patients treated with radical cystectomy followed by adjuvant chemotherapy in two independent datasets

**Figure S1.** Histologic similarity of patient tumors and patient-derived xenografts

**Figure S2.** CDC25C is over-expressed in basal tumor subtypes of human bladder cancer

**Table S1.** Baseline patient and tumor characteristics according to ability of engraftment of patient-derived xenografts

|  |  | **Engraftment (n=69 injected)** | | |
| --- | --- | --- | --- | --- |
| **Characteristic** | **Total** | **Positive** | **Negative** | ***P*-value** |
| Number of patients | 71 | 42 (60.9%) | 27 (39.1%) |  |
| Mean age ± SD (years) | 69.5 ± 10.7 | 69.7 ± 11.9 | 70.1 ± 9.1 | 0.89 |
| Sex |  |  |  |  |
| Male | 50 (70.4%) | 31 (73.8%) | 17 (62.9%) | 0.34 |
| Female | 21 (29.6%) | 11 (26.2%) | 10 (37.0%) |  |
| Race |  |  |  |  |
| Caucasian | 51 (71.8%) | 30 (71.4%) | 19 (70.4%) | 0.72 |
| African-American | 12 (16.9%) | 6 (14.9%) | 6 (22.2%) |  |
| Asian | 5 (7%) | 4 (9.5%) | 1 (3.7%) |  |
| Hispanic | 1 (1.4%) | 1 (2.9%) | 0 |  |
| Other / Unknown | 2 (2.8%) | 1 (2.9%) | 1 (3.7%) |  |
| **Pathology** |  |  |  |  |
| Pathologic Tumor Stage |  |  |  |  |
| Ta, T1, Tis | 9 (12.7%) | 4 (9.5%) | 5 (18.5%) | 0.64 |
| T2 | 11 (15.5%) | 6 (14.3%) | 4 (14.8%) |  |
| T3 | 40 (56.3%) | 24 (57.1%) | 15 (55.6%) |  |
| T4 | 11 (15.5%) | 8 (19%) | 3 (11.1%) |  |
| Pathologic Nodal Stage |  |  |  |  |
| N0 | 42 (59.2%) | 25 (59.5%) | 16 (59.3%) | 0.29 |
| N1 | 7 (9.9%) | 6 (14.3%) | 1 (3.7%) |  |
| N2 | 22 (31%) | 11 (26.2%) | 10 (37%) |  |
| Histology |  |  |  |  |
| Urothelial / Papillary | 57 (80.3%) | 33 (78.6%) | 22 (81.5%) | 0.64 |
| Squamous differentiation | 9 (12.7%) | 5 (11.9%) | 4 (14.8%) |  |
| Other | 5 (7%) | 4 (9.5%) | 1 (3.7%) |  |
| Positive margin | 16 (22.5%) | 10 (23.8%) | 6 (22.2%) | 0.88 |
| Tumor multifocality | 22 (31%) | 13 (31%) | 9 (33.3%) | 0.84 |
| **Additional Treatment** |  |  |  |  |
| Chemotherapy | 28 (39.4%) | 17 (40.5%) | 10 (37%) | 0.65 |
| Pre-operative | 5 (7%) | 5 (11.9%) | 0 |  |
| Post-operative | 18 (25.4%) | 10 (23.8%) | 7 (25.9%) |  |
| Pre- and post-operative | 5 (7%) | 2 (4.8%) | 3 (11.1%) |  |
| **Tumor Cell Classification** |  |  |  |  |
| Basal (triple-pos.) | 28 (39.4%) | 21 (50%) | 7 (26%) | 0.52 |
| Intermediate (double-pos.) | 9 (12.7%) | 4 (9.5%) | 3 (11.1%) |  |
| Differentiated (single-pos.) | 19 (26.7%) | 15 (35.7%) | 4 (14.8%) |  |

Of 71 tumors obtained at radical cystectomy, 69 contained sufficient tissue for implantation in NOD/SCID mice. *P*-value determined using χ2 tests between PDX engrafters (positive) and non-engrafters (negative). “Other” histology includes small cell and sarcomatoid subtypes.

**Table S2.** Tumor cell subpopulations in patient specimens and over successive generations of patient-derived xenografts

| **Patient / PDX** | **Basal** | **Intermediate** | **Differentiated** | **Fully Differentiated** |
| --- | --- | --- | --- | --- |
| Patient 1067 | 0% | 0% | 68% | 32% |
| G0 | 0% | 4.6% | 77% | 18% |
| G1 | 0% | 0.6% | 1.0% | 98% |
| G2 | 0% | 51% | 18% | 32% |
| Patient 1069 | 0.4% | 9.2% | 39% | 51% |
| G0 | 0.5% | 2.2% | 34% | 64% |
| Patient 1071 | 4% | 2.1% | 3.4% | 91% |
| G0 | 0.6% | 0.2% | 13% | 87% |
| Patient 1072 | 0.1% | 1.8% | 31% | 67% |
| G0 | 0% | 1.1% | 48% | 51% |
| G1 | 0% | 2.1% | 6.5% | 91% |
| G2 | 0.1% | 11% | 20% | 69% |

Frequencies of each cell population were determined using flow cytometric isolation. Patient ### denotes primary bladder tumor obtained at surgery; G0 = initial mouse xenograft; G1 = first generation of passage; G2 = second generation of passage.

**Table S3.** Categorization of patient-derived bladder tumor cell lines by flow cytometry

| **Cell line** | **%TP** | **%DP** | **%SP** | **%TN** |
| --- | --- | --- | --- | --- |
| 277 clone 7 | 0% | 0% | 1.1% | 98.9% |
| 289 clone 2 | 0% | 0% | 3.3% | 96.6% |
| 291 clone 6 | 0% | 0% | 44.8% | 55.2% |
| 302 clone 2 | 0% | 0% | 28.3% | 71.7% |
| 303 clone 2 | 0% | 0% | 20.4% | 79.5% |
| 9373 | 0% | 0% | 33.8% | 66.1% |
| 6893 | 0% | 54.1% | 43.1% | 2.8% |
| 5926 clone 1 | 16.4% | 10.7% | 17.7% | 55.1% |
| 5926 clone 2 | 66% | 7.8% | 6.3% | 19.7% |

Tumor cell subpopulations are indicated as the percentage of total bladder tumor cells.

**Table S4.** Genes correlating or anti-correlating with bladder tumor cell differentiation

| **Gene** | **Correlation** |
| --- | --- |
| ABCF3 | -0.6426 |
| ADK | 0.5106 |
| ADNP | -0.5124 |
| AKR1A1 | -0.5028 |
| AKR7A2 | -0.5516 |
| AKR7A3 | -0.5820 |
| AKT1 | 0.5005 |
| ALG9 | 0.5457 |
| ANAPC5 | 0.5129 |
| APM-1 | -0.5106 |
| ARMC5 | -0.5686 |
| ARNT | -0.5159 |
| ATP6V0B | -0.6666 |
| ATP6V1A | -0.5039 |
| BCL2L13 | -0.6203 |
| BCL3 | -0.5091 |
| BLVRA | 0.5072 |
| BMF | -0.5442 |
| BTF3L4 | -0.5814 |
| C10ORF61 | 0.5088 |
| C12ORF65 | 0.5557 |
| C1ORF56 | -0.6490 |
| C1ORF69 | -0.5088 |
| C3ORF17 | 0.5587 |
| C6ORF167 | 0.5523 |
| C6ORF173 | 0.5352 |
| C9ORF37 | -0.5536 |
| CCDC25 | 0.6192 |
| CCDC51 | -0.5319 |
| CDC25C | 0.5114 |
| CDC34 | -0.5044 |
| CDH1 | -0.5192 |
| CDK2 | -0.5759 |
| CENTG2 | 0.5147 |
| CHERP | -0.5320 |
| CHMP1B | -0.5350 |
| CHST7 | 0.5068 |
| CLMN | -0.5246 |
| CLPTM1 | -0.5696 |
| CMPK1 | -0.5456 |
| CNIH4 | 0.5393 |
| CNN3 | -0.6042 |
| COL4A1 | 0.5015 |
| COMMD9 | 0.5120 |
| COQ6 | 0.5000 |
| COX7A2L | -0.5088 |
| CPLX1 | -0.6993 |
| CTDSPL2 | -0.5391 |
| CUGBP1 | -0.5223 |
| CXORF64 | 0.5346 |
| CYB561D1 | -0.5843 |
| CYLN2 | 0.5056 |
| DARS | 0.5266 |
| DCTN3 | 0.5031 |
| DDX54 | 0.5168 |
| DDX58 | 0.5329 |
| DLAT | -0.5747 |
| DNAL1 | 0.6165 |
| DNAL4 | -0.6042 |
| DOLPP1 | -0.6034 |
| DYRK1A | -0.6010 |
| EAF2 | -0.6275 |
| EPN3 | -0.5142 |
| ERBB2 | -0.5049 |
| FAM119B | -0.5787 |
| FAM127B | 0.5082 |
| FAM89B | -0.5372 |
| FER | 0.5513 |
| FLJ11235 | -0.5101 |
| FLJ45248 | -0.5437 |
| FNBP1L | -0.5040 |
| FOXQ1 | -0.5155 |
| FST | 0.5083 |
| FUNDC1 | 0.5429 |
| FZD1 | 0.5514 |
| GGA2 | -0.5427 |
| GOLM1 | -0.5443 |
| GRAMD3 | -0.5269 |
| HARS | 0.5474 |
| HBA2 | -0.5589 |
| HEATR1 | 0.5450 |
| HIAT1 | -0.5196 |
| HIST2H2AA4 | -0.5391 |
| HPS4 | 0.5211 |
| HS.116157 | 0.5466 |
| HS.127279 | 0.5240 |
| HS.132394 | 0.5465 |
| HS.40289 | -0.5710 |
| HS.430851 | -0.5050 |
| HS.462257 | 0.5117 |
| HS.47141 | 0.5246 |
| HS.542937 | 0.6278 |
| HS.569204 | 0.5344 |
| HS.571403 | 0.6377 |
| HS2ST1 | 0.5212 |
| HSD17B3 | 0.6177 |
| IDH3A | 0.5091 |
| IL1F5 | -0.6571 |
| ILDR1 | -0.5430 |
| INPP5E | 0.6057 |
| ITPKC | -0.5326 |
| JUN | -0.5644 |
| KATNAL1 | 0.5697 |
| KHDC1L | 0.5238 |
| KIAA1522 | -0.5433 |
| KLF5 | -0.5160 |
| KRT17P3 | -0.5780 |
| L3MBTL | 0.5586 |
| L3MBTL4 | 0.5100 |
| LZTFL1 | -0.5510 |
| MAP4K2 | -0.5451 |
| MAPK8IP3 | 0.5222 |
| MBTPS1 | -0.5940 |
| MCOLN3 | 0.5164 |
| MEIS2 | -0.5364 |
| MGC61598 | -0.5003 |
| MIR1909 | 0.5301 |
| MLL4 | -0.5314 |
| MORC2 | -0.6175 |
| MRPL15 | 0.5548 |
| MSRB3 | 0.5491 |
| MUT | -0.5526 |
| MYH14 | -0.5427 |
| NAT5 | -0.5372 |
| NDRG2 | -0.5868 |
| NIPSNAP3A | -0.5285 |
| NKPD1 | -0.5168 |
| NOL6 | 0.5583 |
| NR2F6 | -0.5349 |
| NUDT5 | 0.6028 |
| OGDH | 0.5046 |
| PARP16 | -0.5326 |
| PARP2 | 0.5330 |
| PDHA1 | 0.5495 |
| PDRG1 | -0.5280 |
| PEF1 | -0.5626 |
| PELP1 | 0.5365 |
| PEX1 | -0.5038 |
| PEX11B | -0.5021 |
| PEX5 | -0.5364 |
| PFDN6 | -0.5032 |
| PFKP | 0.6255 |
| PHKA1 | 0.6312 |
| PIGF | -0.5757 |
| PLEKHB1 | -0.5503 |
| PNRC2 | -0.5902 |
| POLD3 | -0.5494 |
| POLR3C | 0.5194 |
| POLR3G | 0.6679 |
| POLR3K | 0.5036 |
| PPP1CB | -0.5400 |
| PRKCDBP | 0.6153 |
| PROC | 0.5191 |
| PRR15L | -0.6091 |
| PRUNE | -0.5428 |
| PTPRH | -0.5085 |
| PVRL2 | -0.5597 |
| PWWP2 | -0.5921 |
| PWWP2B | -0.6005 |
| RAB11A | -0.5007 |
| RAB11FIP5 | -0.5203 |
| RAB1A | -0.5295 |
| RAB27A | 0.5151 |
| RBM47 | -0.6599 |
| RNF11 | -0.5154 |
| RNF41 | 0.6352 |
| RP5-1022P6.2 | -0.5902 |
| RP9P | 0.5324 |
| RPL14 | 0.5594 |
| RPL5 | 0.6125 |
| RPL7A | 0.6469 |
| RPP25 | -0.5331 |
| RPRD1A | -0.6310 |
| RRAGB | -0.5523 |
| RTN3 | -0.5267 |
| RWDD4A | -0.6593 |
| SERTAD1 | -0.6269 |
| SETD8 | 0.5515 |
| SFRS2B | -0.5144 |
| SFT2D3 | -0.6052 |
| SGCB | 0.5509 |
| SIRT2 | -0.5171 |
| SLC35D2 | 0.6430 |
| SLC35F3 | 0.5432 |
| SLC44A3 | -0.5433 |
| SLMO1 | 0.5191 |
| SNF8 | 0.6127 |
| SNX16 | -0.5228 |
| SPEG | 0.5130 |
| SSH2 | -0.6844 |
| SSR1 | -0.5171 |
| SUPT5H | -0.5315 |
| SUV420H1 | -0.5090 |
| TBC1D7 | -0.5324 |
| TCEA1 | -0.5018 |
| TDRD6 | 0.5996 |
| TLN1 | 0.5747 |
| TM7SF2 | -0.5983 |
| TMEM184A | -0.5748 |
| TMEM59 | -0.5764 |
| TMEM8B | 0.5882 |
| TOB1 | -0.6211 |
| TRIM35 | -0.5947 |
| TSPAN6 | -0.5105 |
| TTTY6 | 0.5452 |
| TUBB2C | -0.5427 |
| TUFT1 | -0.5037 |
| UBC | -0.5206 |
| UBE2G2 | 0.5029 |
| UBE2K | 0.5798 |
| USP6NL | -0.6104 |
| VASN | -0.5440 |
| VPS13C | -0.5092 |
| VPS37B | -0.5869 |
| VPS52 | 0.5121 |
| VSIG2 | -0.5195 |
| WIPI2 | -0.5805 |
| WWC3 | -0.5090 |
| YARS | 0.5813 |
| YTHDF2 | -0.5185 |
| ZCCHC17 | -0.6418 |
| ZFR | -0.5066 |
| ZNF322B | -0.5140 |
| ZNF701 | -0.5580 |

Correlation coefficients were determined using Pearson correlation analysis of microarray gene expression values and numerical values corresponding to basal (triple-positive=3), intermediate (double-positive=2), differentiated (single-positive=1) and fully differentiated (triple-negative=0) tumor cell subpopulations. Genes with correlation coefficients ≥0.5 (absolute value) were selected for further analysis.

**Table S5.** Immunohistochemical staining for CDC25C in a bladder cancer tissue microarray

| **Sample** | **Histology** | **Grade** | **Stage** | **IHC** |
| --- | --- | --- | --- | --- |
| 1 | Normal bladder tissue | - | - | Negative |
| 2 | Normal bladder tissue | - | - | Negative |
| 3 | Normal bladder tissue | - | - | Negative |
| 4 | Normal bladder tissue | - | - | Negative |
| 5 | Normal bladder tissue | - | - | Negative |
| 6 | Normal bladder tissue | - | - | Negative |
| 7 | Normal bladder tissue | - | - | Negative |
| 8 | Normal bladder tissue | - | - | Negative |
| 9 | Normal bladder tissue | - | - | Negative |
| 10 | Normal bladder tissue | - | - | Minimal |
| 11 | Transitional cell carcinoma | 1 | I | Negative |
| 12 | Transitional cell carcinoma | 2 | I | Negative |
| 13 | Transitional cell carcinoma | 1 | I | Minimal |
| 14 | Transitional cell carcinoma | 1 | I | Minimal |
| 15 | Transitional cell carcinoma | 2 | I | Minimal |
| 16 | Transitional cell carcinoma | 2 | I | Minimal |
| 17 | Transitional cell carcinoma | 2 | I | Minimal |
| 18 | Transitional cell carcinoma | 2 | I | Minimal |
| 19 | Transitional cell carcinoma | 2 | I | Intermediate |
| 20 | Transitional cell carcinoma | 2 | I | Intermediate |
| 21 | Transitional cell carcinoma | 2 | I | Intermediate |
| 22 | Transitional cell carcinoma | 3 | I | Intermediate |
| 23 | Transitional cell carcinoma | 2 | I | High |
| 24 | Transitional cell carcinoma | 2 | I | High |
| 25 | Transitional cell carcinoma | 2 | II | Negative |
| 26 | Transitional cell carcinoma | 2 | II | Minimal |
| 27 | Transitional cell carcinoma | - | II | Minimal |
| 28 | Transitional cell carcinoma | 2 | II | Minimal |
| 29 | Transitional cell carcinoma | 1 | II | Minimal |
| 30 | Transitional cell carcinoma | 1 | II | Minimal |
| 31 | Transitional cell carcinoma | 2 | II | Intermediate |
| 32 | Transitional cell carcinoma | 1 | II | Intermediate |
| 33 | Transitional cell carcinoma | 2 | II | Intermediate |
| 34 | Transitional cell carcinoma | 2 | II | Intermediate |
| 35 | Transitional cell carcinoma | 3 | II | Intermediate |
| 36 | Transitional cell carcinoma | 3 | II | Intermediate |
| 37 | Transitional cell carcinoma | 3 | II | Intermediate |
| 38 | Transitional cell carcinoma | 2 | II | High |
| 39 | Squamous cell carcinoma | 3 | III | Minimal |
| 40 | Transitional cell carcinoma | 1 | III | Minimal |
| 41 | Transitional cell carcinoma | 1 | III | Intermediate |
| 42 | Transitional cell carcinoma | 2 | III | Intermediate |
| 43 | Transitional cell carcinoma | 2 | III | Intermediate |
| 44 | Squamous cell carcinoma | 2 | III | Intermediate |
| 45 | Squamous cell carcinoma | 2 | III | Intermediate |
| 46 | Adenocarcinoma | 3 | III | Intermediate |
| 47 | Transitional cell carcinoma | 2 | III | High |
| 48 | Transitional cell carcinoma | 2 | III | High |
| 49 | Transitional cell carcinoma | 2 | III | High |
| 50 | Transitional cell carcinoma | 1 | IV | Intermediate |

IHC staining was categorized as follows: “Negative” = rare positive cells, <5%; “Minimal” = <25% highly positive cells or majority of cells with <25% stained nucleus; “Intermediate” = 25-75% highly positive cells or majority of cells with 25-75% stained nucleus; “High” = >75% highly positive cells.

**Table S6.** Meta-analysis of multivariate hazard ratio of CDC25C expression accounting for tumor and nodal staging for patients treated with radical cystectomy followed by adjuvant chemotherapy in two independent datasets

| **Data set** | **N** | **HR** | **Lower 95%** | **Upper 95%** |
| --- | --- | --- | --- | --- |
| MD Anderson | 16 | 0.331 | 0.096 | 0.829 |
| Chungbuk | 22 | 1.368 | 0.433 | 4.520 |
| **Cumulative** | **38** | **0.388** | **0.161** | **0.616** |

HR, hazard ratio; Lower and upper 95% denote 95% confidence interval.

**Figure S1. Histologic similarity of patient tumors and patient-derived xenografts** Samples were assessed for tumor architecture using hematoxylin and eosin staining at 10x, 20x and 40x magnification. Shown is one representative pair of samples.

**Figure S2. CDC25C is over-expressed in basal tumor subtypes of human bladder cancer**

CDC25C gene expression was measured in the MD Anderson Cancer Center (MDACC) data set of Choi et al. (*9*) and Lund University Hospital data set of Sjodahl et al. (*13*). Gene expression was determined based on probeset signal intensity values that were median-normalized across each respective data set and log_2_-transformed. Basal (n=19), luminal (n=21) and p53-like (n=13) subtypes were calculated from the MDACC data as previously described (*9*) (**A, left**). Genomically unstable (n=85), SCC-like (n=29), urobasal A (n=131) and B (n=20) and infiltrated (n=43) subtypes were provided in the Lund data set (**B, left**). Based on Aine et al (*13*), luminal and p53-like subtypes were combined into the “Luminal” subtype (**A, right**). In the Lund data set the “Basal” subtype was comprised by SCC-like and Urobasal B tumors, while the “Luminal” subtype consisted of Genomically unstable and Urobasal A tumors (**B, right**). Infiltrated tumors were excluded from this analysis given the mixed basal/luminal expression patterns found in this subtype. Data represent mean ± standard error of mean. *P*-values were determined using Student’s *t*-test between “Basal” and “Luminal” tumor subtypes. * denotes *P* ≤ 0.05, *** denotes *P* ≤ 0.0001.
