# Supplementary figures and images for: Basal Tumor Cell Isolation and Patient-Derived Xenograft Engraftment Identify High-Risk Clinical Bladder Cancers
Source: Sci Rep. 2016 Oct 24;6:35854. doi: 10.1038/srep35854 (PMC5075783; doi:10.1038/srep35854)

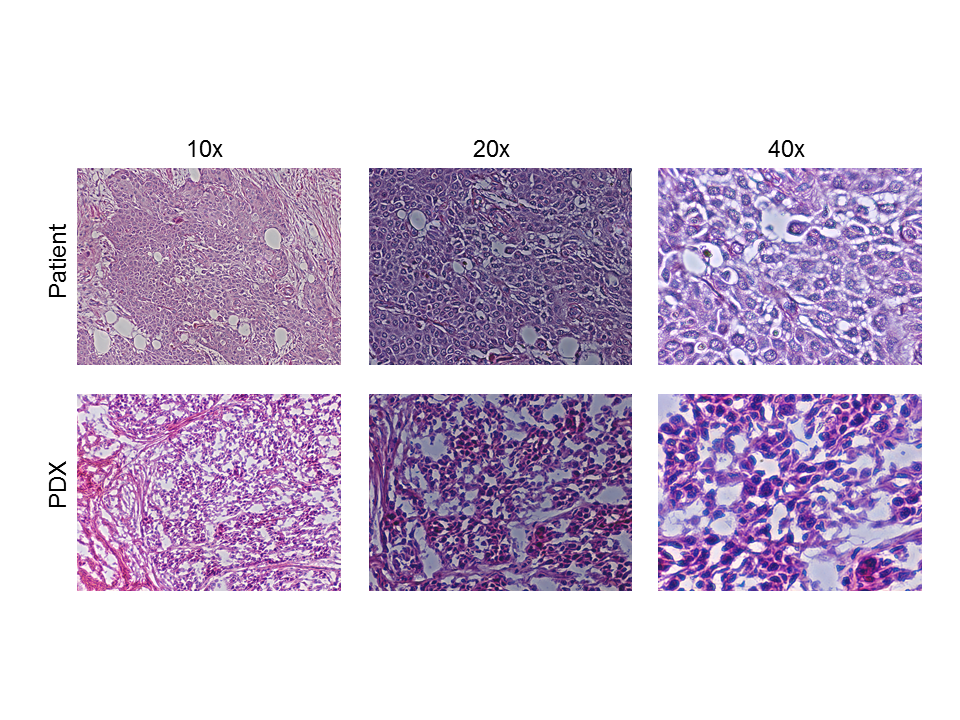

Supplement: Supplementary Figure S1 [file srep35854-s2.tiff]

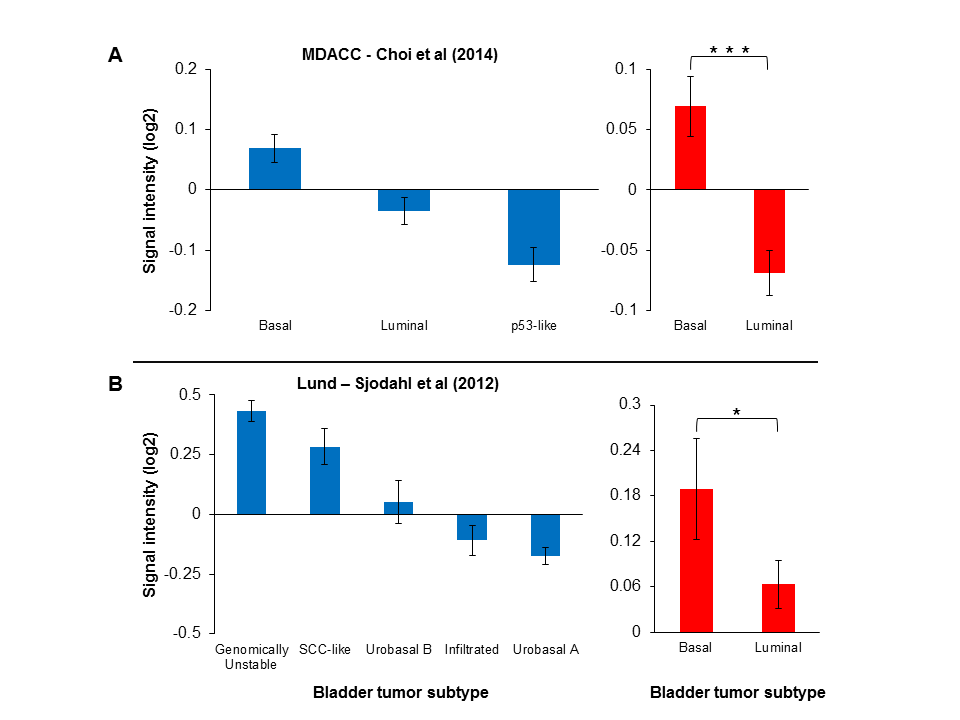

Supplement: Supplementary Figure S2 [file srep35854-s3.tiff]
